# Comprehensive Review on Fruit of *Terminalia chebula*: Traditional Uses, Phytochemistry, Pharmacology, Toxicity, and Pharmacokinetics

**DOI:** 10.3390/molecules29235547

**Published:** 2024-11-24

**Authors:** Changjian Wang, Hongfei Zhang, Xiangdong Wang, Xinyue Wang, Xinru Li, Cuiying Li, Yuefei Wang, Min Zhang

**Affiliations:** 1 State Key Laboratory of Chinese Medicine Modernization, Tianjin Key Laboratory of TCM Chemistry and Analysis, Tianjin University of Traditional Chinese Medicine, Tianjin 301617, China; wangchangjian23@163.com (C.W.); 17866715661@163.com (H.Z.); xiangdongblue@163.com (X.W.); wangxy000821@163.com (X.W.); 16629233345@163.com (X.L.); lcying246@163.com (C.L.); 2 Haihe Laboratory of Modern Chinese Medicine, Tianjin 301617, China

**Keywords:** Chebulae Fructus, phytochemistry, pharmacology, traditional medicine, pharmacokinetics

## Abstract

*Terminalia chebula* Retz., known for its dried fruit, namely Chebulae Fructus, is a medicinal plant with a long-standing global reputation, which was initially recognized for its therapeutic properties during the Jin Dynasty. This review consolidates current knowledge on the traditional uses, phytochemistry, pharmacological properties, toxicity, and pharmacokinetics of Chebulae Fructus, highlighting its clinical significance and the promising therapeutic potential of its compounds. To date, studies have identified approximately 149 compounds within the plant, including tannins, phenolic acids, lignans, triterpenes, flavonoids, and volatiles. These compounds confer a broad spectrum of biological activities in vitro and in vivo, such as antioxidant, anti-inflammatory, antiviral, anticancer, antibacterial, hepatoprotective, nephroprotective, neuroprotective, and anti-diabetic, some of which are already integrated into clinical practice. However, despite substantial advancements, considerable gaps remain in understanding the complete mechanisms of action, pharmacokinetics, and safety profiles of its extracts and compounds. This paper advocates for enhanced focus on these areas to fully elucidate the therapeutic capacities and facilitate the clinical application of Chebulae Fructus. This comprehensive analysis not only reinforces the ethnopharmacological significance of Chebulae Fructus but also lays a foundation for future pharmacological explorations.

## 1. Introduction

**Figure 1:**
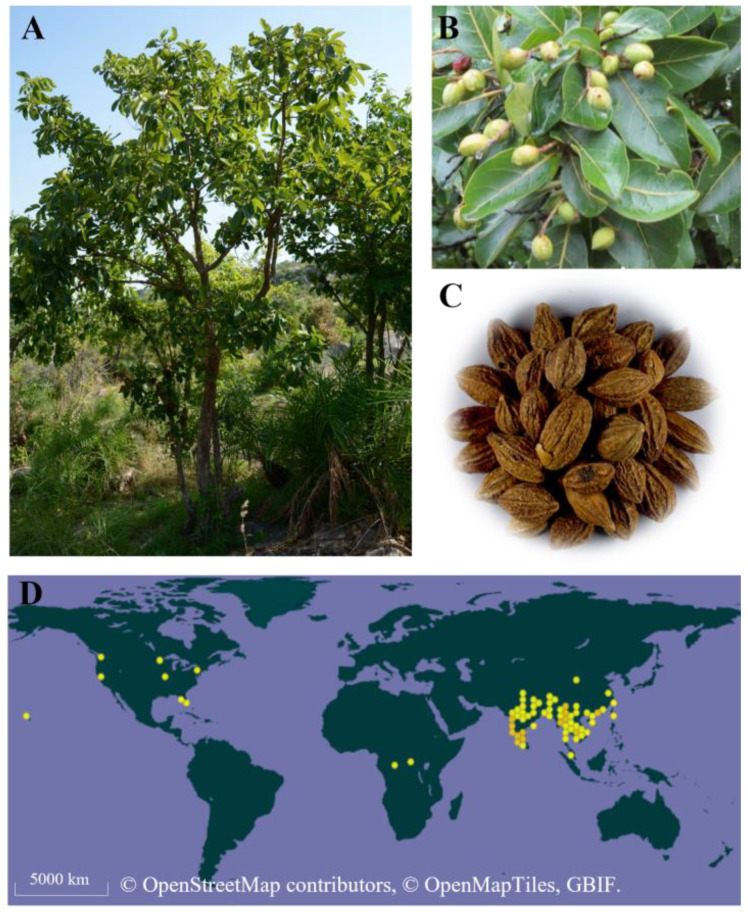
A whole *Terminalia chebula* Retz. plant (**A**); *T. chebula* fruits (**B**); *T. chebula* dried fruits (**C**); and a *T. chebula* global distribution map of the world (**D**) (reprinted from https://www.gbif.org/species/3189388, accessed on 1 October 2024).

*Terminalia chebula* Retz. (*T. chebula* Retz), native to South and Southeast Asia, is highly regarded in both Tibetan and Ayurvedic medicine [1]. *T. chebula* is a medium-to-large-sized tree that belongs to the Combretaceae family (Figure 1A,B). Known as Chebulae Fructus (CF), its dried fruit is extensively used, especially in China, Nepal, India, Myanmar, Sri Lanka, Thailand, and Bangladesh, among others [2] (Figure 1C,D). CF is celebrated as the king of Tibetan medicines and is consistently named first in the Ayurvedic Materia Medica. It has gained popularity as a nutraceutical food and dietary supplement [3]. Owing to its significant clinical efficacy, CF has emerged as one of the most popular herbal medicines and is widely employed in treating bronchitis, coughs, and various diseases associated with the digestive system [4,5].

In recent years, the remarkable therapeutic and nutritional values of CF have gained widespread attention, prompting extensive and significant scholarly research. Phytochemical studies have yielded over 149 identified compounds in various parts of CF, including monoterpenes, sesquiterpenes, diterpenes, flavonoids, phenols, phenylpropanoids, diarylheptanoids, aromatics, fatty acids, polysaccharides, steroids, organic acids, alkaloids, fatty alcohols, and so on. The distinctive essence and healing properties of CF are primarily attributed to its volatile components, such as monoterpenes and sesquiterpenes. Recent pharmacological studies have revealed that CF extracts and isolated compounds possess a broad spectrum of biological activities, including antioxidant, anti-inflammatory, antiviral, anticancer, antibacterial, hepatoprotective, nephroprotective, neuroprotective, and anti-diabetic effects. Many of these properties have long been recognized in traditional folk medicine and subsequently evolved into important pharmaceutical treatments.

This paper refers to the literature on *T. chebula* Retz published before October 2024 from electronic databases such as SciFinder, PubMed, Web of Science, Science Direct, and Google Scholar. Retrieval was based on the following keywords: “*T. chebula* Retz”, “Chebulae Fructus”. Apart from these, we also consulted the *Chinese Pharmacopoeia* (2020) and related ancient traditional Chinese medicine books to further supplement the content of the article. Currently, extensive research and continual updates in the field have made a comprehensive understanding of CF challenging. In light of these ongoing advancements, which have uncovered new compounds and biological activities, this article aims to offer a comprehensive review of the medicinal properties of CF. We delve deeply into its value and potential, aiming to meticulously summarize the latest research progress. Our review differs from that of Nigam et al. in several critical respects [1]. First, it offers a more comprehensive and systematic summary of the chemical constituents found in CF. Moreover, we have broadened the scope of our review to encompass recent developments in traditional uses and pharmacokinetic research. Additionally, our toxicity and pharmacological analysis integrates the most recent literature, thus providing contemporary insights into the field. We strive to equip researchers with the most current and insightful information, laying a scientific foundation for further exploration and utilization of CF. Additionally, this review outlines potential research directions and new prospects for this botanical entity, offering a strategic framework for future investigations.

## 2. Traditional Uses

The fruits of *T. chebula* have been utilized as both food and medicine in various Asian countries since ancient times [6]. According to the *Chinese Pharmacopoeia* (2020 Edition), *T. chebula* Retz. or *T. chebula* Retz. var. tomentella Kurt refers to the dried mature fruits of the plant. They are characterized by bitter and sour flavors, neutral properties, and associations with the lung and large intestine meridians. The fruits are reputed to astringe the lungs and intestines, and benefit the throat, being prescribed for chronic diarrhea, persistent dysentery, ceaseless coughing, throat pain, voice loss, rectal prolapse, blood in the stool, and lung deficiency leading to wheezing and coughing.

In both Ayurveda and Siddha medical systems, the fruits of *T. chebula* are employed in treating a diverse spectrum of health conditions, including chronic diarrhea, gastroenteritis, constipation, malabsorption syndrome, asthma, ulcers, dyspnea, dyspepsia, hemorrhoids, cough, candidiasis, hepatomegaly, urinary discharge, skin diseases, memory loss, epilepsy, cardiovascular diseases, diabetes, anorexia, as a homeostatic agent, and also as a diuretic, antitussive, and wound healer, as reported in numerous studies [7,8].

Historically, the fruits of *T. chebula* has been used in China for over thousands of years, as documented in the *Grasses and Trees in South China* (Western Jin Dynasty, 266–317 AD). This documentation describes it as a tall tree bearing green-yellow, oval-shaped fruits. Further historical references include the *New Compilation of Materia Medica* from the Tang Dynasty (618–907 AD), which notes its bitter flavor, warmth, and non-toxic nature, noting that imports from India began during this period. The *Illustrated Classics of Materia Medica* (Song Dynasty, 960–1279 AD) identifies Guangzhou province as a prime production area for *T. chebula*, renowned for its smaller, superior quality fruits. This distinction is also well illustrated in the *Classified Materia Medica* (Song Dynasty, 960–1279 AD) from the same dynasty, which differentiates *T. chebula* from similar species. The *Essentials of Materia Medica Distinctions* (Ming Dynasty, 1368–1644 AD) similarly sums up these characteristics and specifies Guangzhou as the authentic geographical source with the best quality. These descriptions are largely adopted in the *Compendium of Materia Medica* (Ming Dynasty, 1368–1644 AD).

Despite the extensive historical documentation of its use and numerous modern pharmacological studies reporting novel effects of CF, the potential toxic and side effects associated with CF continue to be debated [1,7,8]. A comprehensive review of both ancient texts and recent scientific research reveals CF exhibits minimal toxic effects. Nevertheless, further research focusing on its safety profile is essential to determine optimal dosages and identify any contraindications in order to fully harness its medicinal potential.

## 3. Phytochemistry

Like other herbs, the pharmacological activities of CF are primarily attributed to its various chemical compounds. To date, more and more pharmacologically active chemical compounds, such as tannins, phenolic acids, lignans, triterpenoids, flavonoids, and volatiles, have been thoroughly understood and have considerably promoted the extensive application of CF. Sources of the chemical compounds reported in CF have been systematically identified through multiple databases, including SciFinder, PubMed, and Web of Science. It has been found that CF contains at least 149 chemical compounds, which comprise 60 tannins, 28 phenolic acids, 15 lignans, 20 triterpenoids, 6 flavonoids, 9 volatiles, and 11 other compounds, such as carboxylic acids and steroids.

### 3.1. Tannins

**Figure 2:**
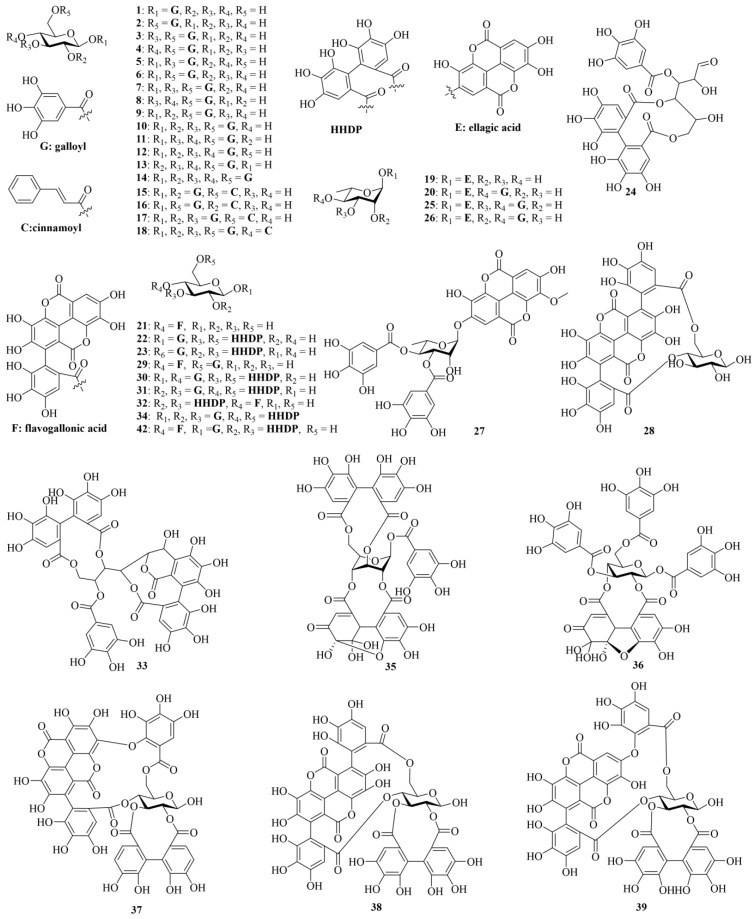

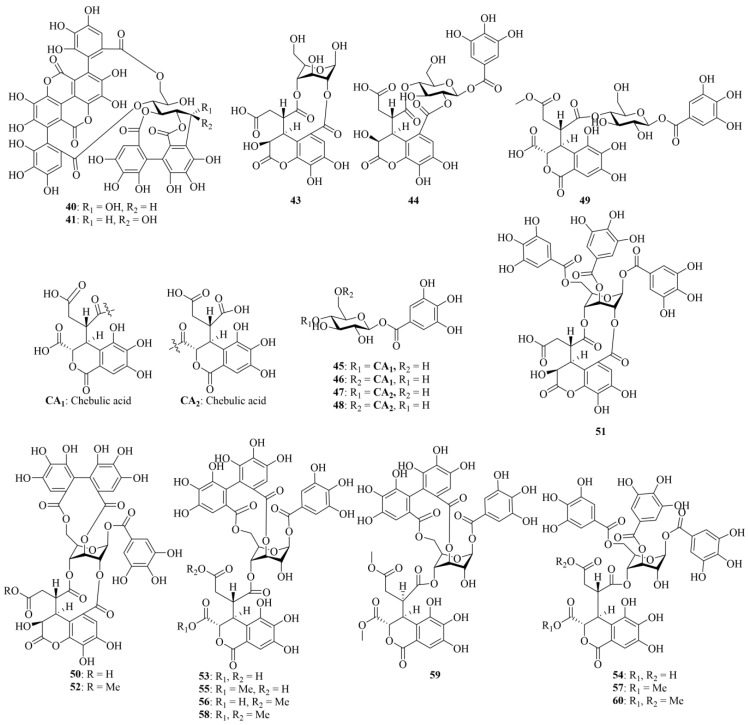
Structures of tannins from Chebulae Fructus.

Tannins are structurally complex polyphenolic compounds found in plants, categorized based on their structure into hydrolyzable tannins, condensed tannins, and complex tannins, with molecular weights typically exceeding 500 Da [9]. Tannins can bind to proteins to produce astringency, which is why chebulic myrobalans have astringent properties. A total of 60 such compounds have so far been reported in CF. The chemical structures of these tannins (**1–60**) are shown in Figure 2. Detailed information about tannins is shown in Table 1. The tannin compounds in CF are predominantly hydrolyzable tannins, which can be divided into three main types based on structural characteristics: gallotannins, ellagitannins, and chebulic ellagitannins.

Gallotannins are formed through ester links between gallic acid (**68**) and glucose or polyols, including compounds such as 1-*O*-galloyl-*β*-D-glucose (**1**), 6-*O*-galloyl-*β*-D-glucose (**2**), 3,6-di-*O*-galloyl-*β*-D-glucose (**3**), and several dimethyl ester derivatives. Ellagitannins are produced by esterification involving hexahydroxydiphenic acid (HHDP) or ellagic acid (**76**) with glucose or rhamnose, for instance, compounds such as eschweilenol C (**19**), 4-*O*-(4″-*O*-galloyl-*α*-L-rhamnosyl) ellagic acid (**20**), and various dimethyl ester derivatives like gemin D (**24**) and tellimagrandin I (**31**) and II (**34**). Chebulic ellagitannins are formed from chebulic acid (**81**) or chebuloyl groups with sugars or polyols, including 2,4-chebuloyl-*β*-D-glucose, chebulanin (44), phyllanemblinin E (**45**) and F (**46**), chebumeinin A (**47**) and B (**48**), various methyl ester derivatives of new chebulagic acid (**50**), and dimethyl esters of new chebuloylgallic acids. Lee et al. isolated nine hydrolyzable tannins from CF, comprising three previously unidentified and six artifact compounds, along with thirty-nine known ones. The newly identified compounds were characterized by spectroscopic analysis as 1,2,3-tri-*O*-galloyl-6-*O*-cinnamoyl-*β*-D-glucose (**17**), 1,2,3,6-tetra-*O*-galloyl-4-*O*-cinnamoyl-*β*-D-glucose (**18**), and 4-*O*-(2″,4″-di-*O*-galloyl-α-L-rhamnosyl) ellagic acid (**26**). Additionally, the following artifact compounds were identified: 1′-*O*-methyl neochebulanin (**49**), methyl chebulagate (**52**), 6′-*O*-methyl neochebulagate (**56**), dimethyl neochebulagate (**58**), dimethyl 4′-*epi*-neochebulagate (**59**), and dimethyl neochebulinate (**60**) [10]. Xu et al. developed a method using ultra-performance liquid chromatography coupled with a photodiode array detector to analyze 12 common tannic and phenolic constituents in CF. The total contents of these constituents were quantitatively measured in different parts of CF, with the highest levels found in the exocarp at 148.86 mg/g. During the sunlight-drying process, hydrolyzable tannins (such as punicalagin (**38**), chebulanin (**44**), chebulagic acid (**50**), and chebulinic acid (**51**)) in CF were found to degrade into simpler acids like gallic acid (**68**), ellagic acid (**76**), and chebulic acid (**81**), indicating significant changes in chemical composition due to processing [11].

### 3.2. Phenolic Acids

**Figure 3:**
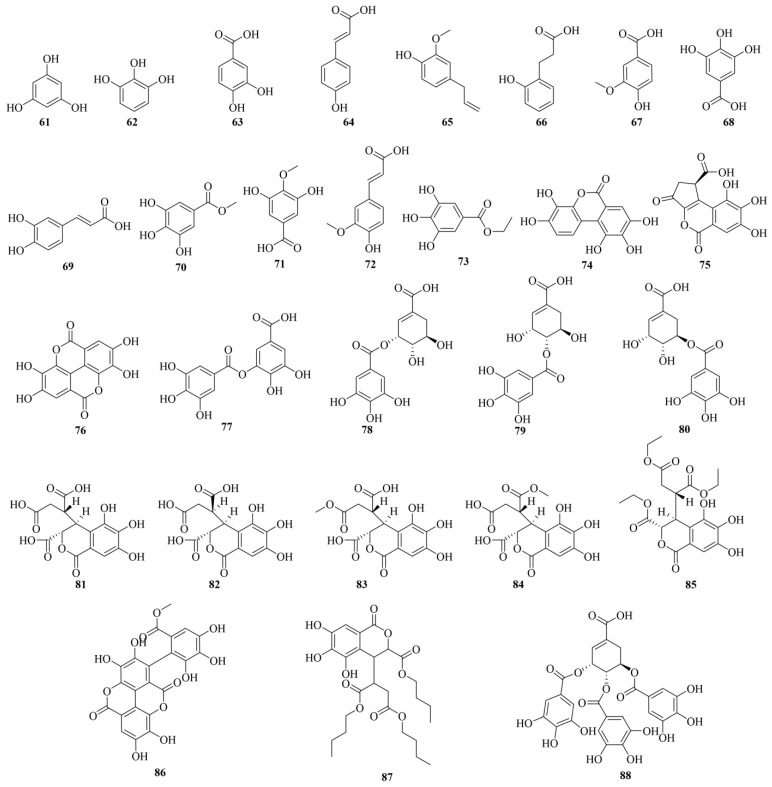
Structures of phenolic acids from Chebulae Fructus.

Phenolic acids represent the most predominant group of bioactive constituents found in various plant sources [12]. To date, 28 compounds have been reported in CF. The chemical structures of phenolic acids (**61–88**) are shown in Figure 3. Information on their identity is shown in Table 1. Phenolic acids are mainly categorized into hydroxybenzoic and hydroxycinnamic acids, which contribute characteristic organoleptic qualities to foods and are associated with numerous health benefits. Hydroxybenzoic acids, derivatives of benzoic acid, feature a seven-carbon atom framework conforming to a C6-C1 structure, exemplified by compounds such as protocatechuic acid (**63**) and gallic acid (**68**). Hydroxycinnamic acids, derivatives of cinnamic acid, possess a C6-C3 structure, with common examples including caffeic acid (**69**) and ferulic acid (**72**). Additionally, a minor group exists outside these categories, such as ellagic acid, which is a dimer of gallic acid (**68**). Tubtimdee et al. utilized a spherical central composite design to optimize the extraction conditions of total phenolics, including gallic acid (**68**) and elagic acid (**76**), from CF using water–ethanol and water–propylene glycol mixtures. The study meticulously adjusted variables such as solvent concentration, temperature, and extraction time to maximize the yield of these specific phenolic compounds [13].

### 3.3. Lignans

**Figure 4:**
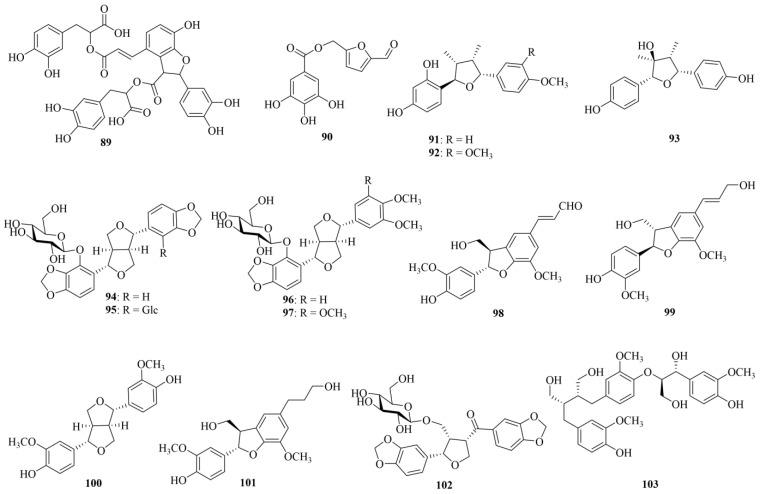
Structures of lignans from Chebulae Fructus.

Lignans are composed of two or more phenylpropanoid (a C6–C3 structure) units. The monomers of lignans primarily consist of cinnamic acid, cinnamyl alcohol, propenyl benzene, and allyl benzene [14]. To date, 15 such compounds have so far been reported in CF. The chemical structures of these lignans (**89–103**) are shown in Figure 4. Detailed information about the lignans is shown in Table 1. The main lignans in CF include lithospermate B (**89**), terminaliate A (**90**), and termitomenin A (**91**).

### 3.4. Triterpenoids

**Figure 5:**
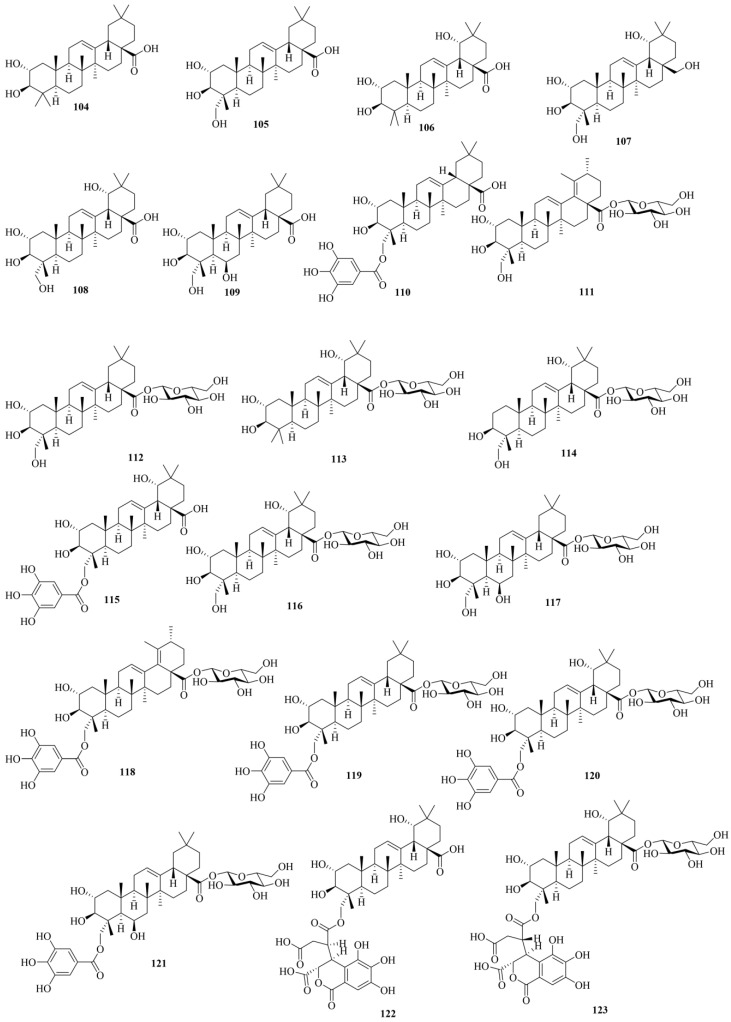
Structures of triterpenoids from Chebulae Fructus.

The triterpenoid compounds in CF primarily consist of pentacyclic triterpenes and their glycosides. Triterpenoids are a class of terpenoid compounds fundamentally comprising a carbon skeleton of 30 carbon atoms, existing in plants either in free form or conjugated with sugars as glycosides or esters. These compounds exhibit a wide range of biological activities. To date, 20 triterpene compounds have been reported in CF. The chemical structures of triterpenes (**104–123**) are shown in Figure 5. Detailed information about triterpenes is shown in Table 1. The main triterpenoids in CF include maslinic acid (**104**), arjunolic acid (**105**), arjunic acid (**106**), and chebupentol (**107**).

### 3.5. Flavonoids

**Figure 6:**
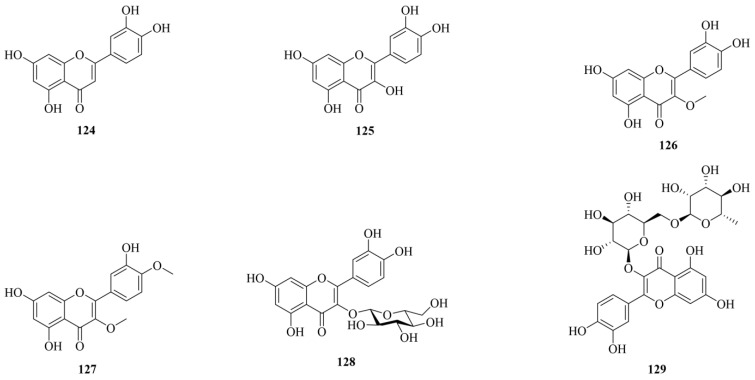
Structures of flavonoids from Chebulae Fructus.

Flavonoid compounds are widely distributed in nature, with the majority being hydroxy-substituted flavonoid derivatives. The flavonoid core can also have methoxy groups or other substituents. To date, 6 such compounds have been reported in CF. The chemical structures of flavonoids (**124–129**) are shown in Figure 6. Information on their identity is shown in Table 1. In CF, the main flavonoids include luteolin (**124**), quercetin (**125**), 3-methoxy quercetin (**126**), 3,4′-dimethoxy quercetin (**127**), isoquercetin (**128**), and rutin (**129**).

### 3.6. Volatiles

**Figure 7:**
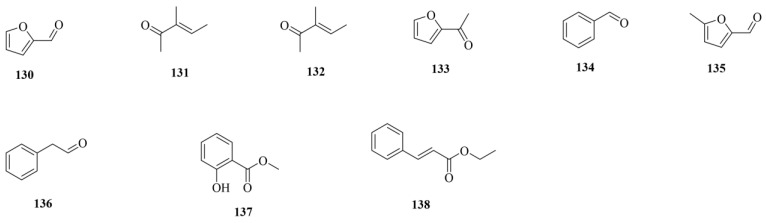
Structures of volatiles from Chebulae Fructus.

Volatiles are a class of small, volatile compounds that act as secondary metabolites. Dattatraya identified nine volatile components in CF through comparison with standards using GC-MS [15]. The chemical structures of these volatiles (**130–138**) are shown in Figure 7. Detailed information about the volatiles is shown in Table 1. These include furfural (**130**), 3-methyl-3-penten-2-one (**131**), 2-cyclopentene-1,4-dione (**132**), 2-acetylfuran (**133**), benzaldehyde (**134**), 5-methylfurfural (**135**), phenylacetaldehyde (**136**), methyl salicylate (**137**), and ethyl cinnamate (**138**).

### 3.7. Other Chemicals

**Figure 8:**
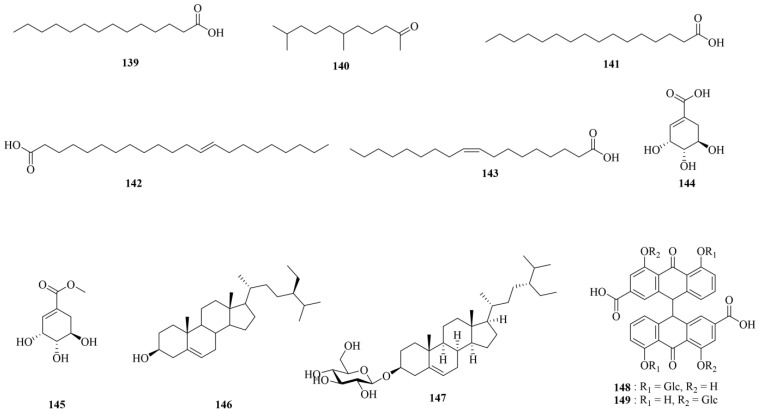
Structures of other chemicals from Chebulae Fructus.

CF comprises various fatty acids, including myristic acid (**139**), 6,10-dimethyl-2-undecanone (**140**), palmitic acid (**141**), 13-docosenoic acid (**142**), and oleic acid (**143**). It also features carboxylic acid compounds like shikimic acid (**144**) and methyl shikimate (**145**). Additionally, it includes *β*-sitosterol (**146**), daucosterol (**147**), sennoside A (**148**), and sennoside B (**149**). The chemical structures of these other chemicals (**139–149**) are shown in Figure 8. Detailed information regarding these other chemicals is shown in Table 1.

**Table 1:** Information regarding compounds from Chebulae Fructus.

NO.	Classification	Compound	Molecular Formula	Ref.
	**Tannins**			
1		1-*O*-galloyl-*β*-D-glucose	C_13_H_16_O_10_	[16]
2		6-*O*-galloyl-*β*-D-glucose	C_13_H_16_O_10_	[10]
3		3,6-di-*O*-galloyl-*β*-D-glucose	C_20_H_20_O_14_	[16]
4		4,6-di-*O*-galloyl-*β*-D-glucose	C_20_H_20_O_14_	[16]
5		1,3-di-*O*-galloyl-*β*-D-glucose	C_20_H_20_O_14_	[10]
6		1,6-di-*O*-galloyl-*β*-D-glucose	C_20_H_20_O_14_	[10]
7		1,3,6-tri-*O*-galloyl-*β*-D-glucose	C_27_H_24_O_18_	[17]
8		3,4,6-tri-*O*-galloyl-*β*-D-glucose	C_27_H_24_O_18_	[10]
9		1,2,6-tri-*O*-galloyl-*β*-D-glucose	C_27_H_24_O_18_	[18]
10		1,2,3,6-tetra-*O*-galloyl-*β*-D-glucose	C_34_H_28_O_22_	[10]
11		1,3,4,6-tetra-*O*-galloyl-*β*-D-glucose	C_34_H_28_O_22_	[10]
12		1,2,3,4-tetra-*O*-galloyl-*β*-D-glucose	C_34_H_28_O_22_	[19]
13		2,3,4,6-tetra-*O*-galloyl-*β*-D-glucose	C_34_H_28_O_22_	[20]
14		1,2,3,4,6-penta-*O*-galloyl-*β*-D-glucose	C_41_H_32_O_26_	[16]
15		1,2-di-*O*-galloyl-6-*O*-cinnamoyl-*β*-D-glucose	C_29_H_26_O_15_	[10]
16		1,6-di-*O*-galloyl-2-*O*-cinnamoyl-*β*-D-glucose	C_29_H_26_O_15_	[10]
17		1,2,3-tri-*O*-galloyl-6-*O*-cinnamoyl-*β*-D-glucose	C_36_H_30_O_19_	[10]
18		1,2,3,6-tetra-*O*-galloyl-4-*O*-cinnamoyl-*β*-D-glucose	C_43_H_34_O_23_	[10]
19		eschweilenol C	C_20_H_16_O_12_	[10]
20		4-*O*-(4″-*O*-galloyl-*α*-L-rhamnosyl) ellagic acid	C_27_H_20_O_16_	[10]
21		terflavin D	C_27_H_20_O_18_	[21]
22		Corilagin	C_27_H_22_O_18_	[17]
23		2,3-(*S*)-HHDP-6-*O*-galloyl-*β*-D-glucose	C_27_H_22_O_18_	[16]
24		gemin D	C_27_H_22_O_18_	[10]
25		4-*O*-(3″,4″-di-*O*-galloyl)-*α*-L-rhamnosyl-ellagic acid	C_34_H_24_O_20_	[10]
26		4-*O*-(2″,4″-di-*O*-galloyl-*α*-L-rhamnosyl) ellagic acid	C_34_H_24_O_20_	[10]
27		3′-*O*-methyl-4-*O*-(3″,4″-di-*O*-galloyl-*α*-L-rhamnopyranosyl) ellagic acid	C_35_H_26_O_20_	[22]
28		Punicalin	C_34_H_22_O_22_	[1]
29		terflavin B	C_34_H_24_O_22_	[21]
30		Tercatain	C_34_H_26_O_22_	[10]
31		tellimagrandin I	C_34_H_26_O_22_	[10]
32		terflavin C	C_41_H_26_O_26_	[21]
33		Casuarinin	C_41_H_28_O_26_	[1]
34		tellimagrandin II	C_41_H_30_O_26_	[16]
35		Geraniin	C_41_H_28_O_27_	[17]
36		Terchebin	C_41_H_30_O_27_	[22]
37		Isoterchebulin	C_48_H_28_O_29_	[23]
38		Punicalagin	C_48_H_28_O_30_	[17]
39		Terchebulin	C_48_H_28_O_30_	[21]
40		punicacortein C	C_48_H_28_O_30_	[17]
41		punicacortein D	C_48_H_28_O_30_	[10]
42		terflavin A	C_48_H_30_O_30_	[1]
43		2,4-chebuloyl-*β*-D-glucopyranoside	C_20_H_20_O_15_	[24]
44		Chebulanin	C_27_H_24_O_19_	[17]
45		phyllanemblinin E	C_27_H_26_O_20_	[10]
46		phyllanemblinin F	C_27_H_26_O_20_	[10]
47		chebumeinin A	C_27_H_26_O_20_	[25]
48		chebumeinin B	C_27_H_26_O_20_	[26]
49		1′-*O*-methyl neochebulanin	C_28_H_28_O_20_	[10]
50		chebulagic acid	C_41_H_30_O_27_	[17]
51		chebulinic acid	C_41_H_32_O_27_	[24]
52		methyl chebulagate	C_42_H_32_O_27_	[10]
53		neochebulagic acid	C_41_H_32_O_28_	[10]
54		neochebulinic acid	C_41_H_34_O_28_	[27]
55		methyl neochebulagate	C_42_H_34_O_28_	[28]
56		6′-*O*-methyl neochebulagate	C_42_H_34_O_28_	[10]
57		1′-*O*-methyl neochebulinate	C_42_H_36_O_28_	[10]
58		dimethyl neochebulagate	C_43_H_36_O_28_	[10]
59		dimethyl 4′-*epi*-neochebulagate	C_43_H_36_O_28_	[10]
60		dimethyl neochebulinate	C_43_H_38_O_28_	[10]
	**Phenolic acids**			
61		Phloroglucinol	C_6_H_6_O_3_	[1]
62		Pyrogallol	C_6_H_6_O_3_	[1]
63		protocatechuic acid	C_7_H_6_O_4_	[1]
64		*p*-coumaric acid	C_9_H_8_O_3_	[1]
65		Eugenol	C_10_H_12_O_2_	[1]
66		melilotic acid	C_9_H_10_O_3_	[1]
67		vanillic acid	C_8_H_8_O_4_	[1]
68		gallic acid	C_7_H_6_O_5_	[16]
69		caffeic acid	C_9_H_8_O_4_	[1]
70		methyl gallate	C_8_H_8_O_5_	[17]
71		4-*O*-methylgallic acid	C_8_H_8_O_5_	[1]
72		ferulic acid	C_10_H_10_O_4_	[1]
73		ethyl gallate	C_9_H_10_O_5_	[1]
74		urolithin M5	C_13_H_8_O_7_	[16]
75		brevifolin carboxylic acid	C_13_H_8_O_8_	[10]
76		ellagic acid	C_14_H_6_O_8_	[17]
77		digallic acid	C_14_H_10_O_9_	[10]
78		3-*O*-galloylshikimic acid	C_14_H_14_O_9_	[29]
79		4-*O*-galloylshikimic acid	C_14_H_14_O_9_	[10]
80		5-*O*-galloylshikimic acid	C_14_H_14_O_9_	[16]
81		chebulic acid	C_14_H_12_O_11_	[10]
82		neochebulic acid	C_14_H_12_O_11_	[30]
83		6′-*O*-methyl chebulate	C_15_H_14_O_11_	[10]
84		7′-*O*-methyl chebulate	C_15_H_14_O_11_	[10]
85		(-)-chebulic acid triethyl ester	C_20_H_24_O_11_	[31]
86		methyl (*S*)-flavogallonate	C_22_H_12_O_13_	[28]
87		tri-*n*-butyl chebulate	C_26_H_36_O_11_	[32]
88		tri-*O*-galloylshikimic acid	C_28_H_22_O_17_	[22]
	**Lignans**			
89		lithospermate B	C_36_H_30_O_16_	[26]
90		terminaliate A	C_13_H_10_O_7_	[10]
91		termitomenin A	C_19_H_22_O_4_	[33]
92		termitomenin B	C_20_H_24_O_5_	[33]
93		termitomenin C	C_18_H_20_O_4_	[33]
94		termitomenin D	C_26_H_28_O_12_	[33]
95		termitomenin E	C_32_H_38_O_18_	[33]
96		termitomenins F	C_27_H_32_O_12_	[34]
97		termitomenins G	C_28_H_34_O_13_	[34]
98		(−)-balanophonin	C_20_H_20_O_6_	[33]
99		dehydrodiconiferyl alcohol	C_20_H_22_O_6_	[28]
100		(+)-pinoresinol	C_20_H_22_O_6_	[19]
101		*rel*-(2*α*,3*β*)-7-*O*-methylcedrusin	C_20_H_24_O_6_	[27]
102		(+)-(7*S*,8*S*,8′*S*)-9-*O*-[*β*-D-glucopyranoyl]asarininone	C_26_H_28_O_12_	[1]
103		sesquimarocanol B	C_30_H_38_O_10_	[25]
	**Triterpenoids**			
104		maslinic acid	C_30_H_48_O_4_	[10]
105		arjunolic acid	C_30_H_48_O_5_	[10]
106		arjunic acid	C_30_H_48_O_5_	[35]
107		chebupentol	C_30_H_50_O_5_	[10]
108		arjungenin	C_30_H_48_O_6_	[10]
109		terminolic acid	C_30_H_48_O_6_	[10]
110		23-*O*-galloylarjunolic acid	C_37_H_52_O_9_	[10]
111		pinfaenoic acid 28-*O*-*β*-D-glucopyranosylester	C_36_H_56_O_10_	[10]
112		arjunglucoside II	C_36_H_58_O_10_	[10]
113		arjunetin	C_36_H_58_O_10_	[10]
114		crataegioside	C_36_H_58_O_10_	[10]
115		23-*O*-galloylarjunic acid	C_37_H_52_O_10_	[10]
116		arjunglucoside I	C_36_H_58_O_11_	[10]
117		chebuloside II	C_36_H_58_O_11_	[10]
118		23-*O*-galloylpinfaenoic acid 28-*O*-*β*-D-glucopyranosyl ester	C_43_H_60_O_14_	[10]
119		23-*O*-galloylarjunolic acid 28-*O*-*β*-D-glucopyranosyl ester	C_43_H_62_O_14_	[10]
120		quercotriterpenoside I	C_43_H_62_O_15_	[10]
121		23-*O*-galloylterminolic acid 28-*O*-*β*-D-glucopyranosyl ester	C_43_H_62_O_15_	[10]
122		23-*O*-4′-*epi*-neochebuloylarjungenin	C_44_H_58_O_16_	[10]
123		23-*O*-neochebuloylarjungenin 28-*O*-*β*-D-glycopyranosyl ester	C_50_H_68_O_21_	[10]
	**Flavonoids**			
124		luteolin	C_15_H_10_O_6_	[1]
125		quercetin	C_15_H_10_O_7_	[1]
126		3-methoxy quercetin	C_16_H_12_O_7_	[1]
127		3,4′-dimethoxy quercetin	C_17_H_14_O_7_	[1]
128		isoquercetin	C_21_H_20_O_12_	[1]
129		rutin	C_27_H_30_O_16_	[1]
	**Volatiles**			
130		furfural	C_5_H_4_O_2_	[15]
131		3-methyl-3-penten-2-one	C_6_H_10_O	[15]
132		2-cyclopentene-1,4-dione	C_5_H_4_O_2_	[15]
133		2-acetylfuran	C_6_H_6_O_2_	[15]
134		benzaldehyde	C_7_H_6_O	[15]
135		5-methylfurfural	C_6_H_6_O_2_	[15]
136		phenylacetaldehyde	C_8_H_8_O	[15]
137		methyl salicylate	C_8_H_8_O_3_	[15]
138		ethyl cinnamate	C_11_H_12_O_2_	[15]
	**Other chemicals**			
139		myristic acid	C_14_H_28_O_2_	[15]
140		6,10-dimethyl-2-undecanone	C_13_H_26_O	[15]
141		palmitic acid	C_16_H_32_O_2_	[15]
142		13-docosenoic acid	C_22_H_42_O_2_	[15]
143		oleic acid	C_18_H_34_O_2_	[15]
144		shikimic acid	C_7_H_10_O_5_	[36]
145		methyl shikimate	C_8_H_12_O_5_	[36]
146		*β*-sitosterol	C_29_H_50_O	[26]
147		sitogluside	C_35_H_60_O_6_	[37]
148		sennoside A	C_42_H_38_O_20_	[38]
149		sennoside B	C_42_H_38_O_20_	[38]

## 4. Pharmacological Properties

**Figure 9:**
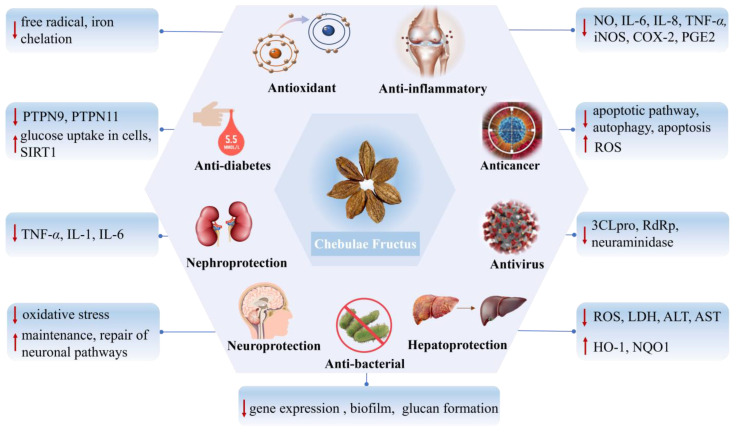
The main pharmacological action of Chebulae Fructus (arrow ↓ means decrease, arrow ↑ means increase).

Many modern pharmacological studies have proven antioxidant, anti-inflammatory, antiviral, anticancer, antibacterial, hepatoprotective, nephroprotective, neuroprotective, anti-diabetic, and other activities of CF (Figure 9 and Table 2).

### 4.1. Antioxidant Activity

Antioxidant activity in CF has been widely studied and CF has shown strong activity. The accumulation of reactive oxygen species (ROS) induces oxidative damage to DNA, oxidizes proteins, and leads to lipid peroxidation and inactivation of antioxidant enzymes within cells. This, in turn, contributes to the development of numerous chronic conditions including cancer, diabetes mellitus, cardiovascular diseases, and atherosclerosis. Consequently, the action mechanisms of numerous synthetic pharmaceuticals involve scavenging free radicals to mitigate oxidative damage, although these drugs often come with detrimental side effects [39]. As an alternative, the intake of natural antioxidants found in various dietary supplements and traditional medicinal practices is recommended [40]. Additionally, there is a growing interest among researchers in phytotherapy to employ medicinal plants with antioxidant properties to guard against oxidative stress. Among the edible fruits in the Himalayan region of India, CF with the highest antioxidant level has been used as a daily dietary supplement among people living in the mountains [40]. CF plays an essential role in regulating oxidative stress and is an important source of daily intake of antioxidant supplements. Many studies have shown that CF promotes health through antioxidation [19,41].

The polyphenolic-rich fraction of CF has been identified as a potential source of natural antioxidants. The antioxidant activity of this fraction was assessed by evaluating its reducing power, total antioxidant capacity, and its ability to scavenge 2,2-diphenyl-1picryl-hydrazyl-hydrate (DPPH) radicals (IC_50_ = 14 μg/mL), nitric oxide radicals (IC_50_ = 30.51 μg/mL), and hydrogen peroxide (IC_50_ = 265.53 μg/mL) under in vitro conditions [19]. The antioxidant properties of a 70% methanol extract from CF were assessed through its effectiveness in neutralizing various free radicals, such as hydroxyl, superoxide, nitric oxide, hydrogen peroxide, peroxynitrite, singlet oxygen, and hypochlorous acid. The study also measured the content of phenols, flavonoids, and ascorbic acid. Findings indicated a linear correlation between the content of phenolic acids and flavonoids and the observed antioxidant activity [41]. In a study comparing the antioxidant activity of water, methanol, and 95% ethanol extracts of CF, results from four distinct chemiluminescence assays demonstrated varying levels of efficacy. Among these, the methanol extract was found to contain the highest concentration of total triterpenoids. Conversely, the water extract exhibited the highest amounts of total phenolics and tannins [42]. A further study demonstrated that the hydroethanol extract of CF exhibited high phenolic content and strong antioxidant activity. The antioxidant activity of a compound can typically be attributed to its electron-donating nature, as seen in functional groups like –OH and –COOH. Therefore, the high content of phenolics in the CF extract suggests that a significant number of –OH groups in the phenolic compounds from CF may be responsible for its antioxidant and anti-inflammatory activities [43]. The scavenging activity of CF against DPPH or ABTS radicals was enhanced in a concentration-dependent manner. This finding suggests that CF could be utilized as an antioxidant without being limited by cytotoxic effects [44]. The polyphenolic extract from CF confers protection against oxidative stress induced by ROS. This protective effect is likely attributed to the phytochemical composition, predominantly polyphenols, present in the extract. Polyphenols, owing to their phenolic hydroxyl groups, are recognized for their efficacy in scavenging free radicals. The findings further indicate a correlation between the concentration of polyphenolic compounds and the enhancement of antioxidant activity [45].

Contemporary pharmacological research has demonstrated that CF primarily exhibits antioxidant properties due to its rich production of phenolcarboxylic and tannic antioxidants, such as chebulagic acid (**50**), chebulinic acid (**51**), gallic acid (**68**), and ellagic acid (**76**). These compounds are highly effective in preventing oxidative stress and inhibiting oxygen- or peroxide-induced reactions [46]. They also mitigate the harmful effects of oxidation in animal tissues, thereby offering significant anti-aging benefits [47]. Chebulagic acid (**50**) and chebulinic acid (**51**), two of the effective compounds identified in CF, are widely recognized for their substantial antioxidant capabilities. These effects are facilitated through conventional antioxidant pathways, including ROS scavenging and iron chelation [48]. Gallic acid (**68**), renowned for its strong antioxidant effects, serves a dual function in the food industry and healthcare. Its robust antioxidant properties allow it to counteract free radicals, alleviate oxidative stress, and safeguard cellular integrity [49]. Additional studies have shown that gallic acid mitigates cyclophosphamide-induced renal toxicity in mice by increasing glutathione levels and boosting the activities of enzymes such as superoxide dismutase (SOD), glutathione peroxidase, and catalase [50]. Ellagic acid (**76**) is notably recognized for its potent antioxidant properties, particularly in scavenging free radicals and modulating antioxidant enzyme activity. Research has established that ellagic acid effectively inhibits lipid peroxidation due to its radical scavenging capabilities, underscoring its potential therapeutic benefits in oxidative stress-related conditions [51]. To sum up, the extracts and compounds of CF showed excellent potential with respect to antioxidant effects and had broad application prospects.

### 4.2. Anti-Inflammatory Activity

The inflammatory response serves as a protective mechanism, activated by the body in response to internal and external injurious stimuli. Its primary function is to eliminate harmful agents and facilitate tissue repair at the affected sites. However, chronic inflammation can lead to self-damage [52]. CF and its chemical compounds exhibit anti-inflammatory properties. These compounds mediate their effects by disrupting arachidonic acid metabolism, suppressing nitric oxide (NO) release, and modulating various signaling pathways. These include the nuclear factor kappa B (NF-κB), mitogen-activated protein kinase (MAPK), extracellular-regulated protein kinase (ERK), toll-like receptor 4 (TLR4), myeloid differentiation primary response protein 88 (MyD88), nuclear factor erythroid-derived 2-related factor 2, (Nrf2), hemeoxygenase-1 (HO-1) pathways, and the activation of the NLRP3 inflammasome.

The current research illustrates that CF effectively alleviated symptoms of atopic dermatitis in a dermatophagoides farinae extract-induced mouse model. This amelioration is achieved through enhancement of the skin barrier, reduction in immune cell infiltration, and suppression of inflammation-associated mediator levels. Moreover, CF aqueous extract and its compounds actively inhibit these mediators by dampening the STAT1/3 and NF-κB signaling pathways [53]. In the study, the authors investigate the potential therapeutic effects of CF on osteoarthritis (OA) incidence, focusing on its impact on IL-1*β*-stimulated chondrocytes and a rat model of monosodium iodoacetate (MIA)-induced OA. The research delineates how CF mitigates oxidative stress and the production of pro-inflammatory cytokines, mediators, and matrix metalloproteinases (MMPs) in vitro. Concurrently, it enhances the expression of proteins related to collagen synthesis. From a mechanistic perspective, the study elucidates that CF interrupts the activation pathways of NF-κB and MAPK by inhibiting the phosphorylation of NF-κB p65 and ERK. Notably, comparable therapeutic effects of CF were confirmed in vivo, reinforcing its potential as a promising intervention for OA [54]. In the referenced investigation, the analgesic and anti-inflammatory properties of ethanolic CF were assessed through several methodologies, including the tail-flick method, Eddy’s hot plate method, and the acetic acid-induced writhing test. Additionally, the in vivo anti-inflammatory effects were evaluated by measuring carrageenan-induced rat paw edema. Comparative analyses demonstrated that the ethanolic extract significantly alleviated the edema in a manner comparable to the effects observed with a standard treatment of diclofenac sodium at a dosage of 10 mg/kg. This study suggests that ethanolic CF possesses notable analgesic and anti-inflammatory activities, warranting further investigation for potential therapeutic applications [43]. The ethyl acetate extract of CF significantly reduced the disease activity index scores and mitigated the weight loss and colonic shortening caused by dextran sodium sulfate. Histomorphological examination of the colon and measurements of oxidative stress revealed improvements following CF treatment in comparison to the group experiencing ulcerative colitis induced by dextran sodium sulfate. Additionally, CF moderated the inflammatory response through modulation of the TLR4/MyD88/NF-κB signaling pathway [55].

In addition, CF and its chemical compounds manifest anti-inflammatory properties by modulating pertinent signaling pathways. CF exhibits robust anti-inflammatory activity and is prevalently utilized in managing various conditions, including arthritis, hypertension, atherosclerosis, diabetic nephropathy, and dry eye syndrome. Key active constituents identified include tannins and phenolic acids.

Specifically, compounds such as corilagin (**22**), chebulanin (**44**), chebulagic acid (**50**), and chebulinic acid (**51**) are cited for their efficacious impact in mitigating arthritis symptoms [56,57]. Their anti-inflammatory activity is mainly achieved through the NF-κB and MAPK signaling pathways [58,59,60]. Studies also showed that 1,2,3,4,6-penta-*O*-galloyl-*β*-D-glucose (**14**) protects against diabetic nephropathy kidney injury by alleviating inflammation and oxidative stress by suppressing the MAPK/NF-κB and ERK/Nrf2/HO-1 signaling pathways [61]. The findings concerning the protective effects of chebulinic acid (**51**) on LPS-induced bone loss suggest its promise as a potential novel therapeutic strategy for bone-related inflammatory diseases. It was observed that chebulinic acid (**51**) notably reduced the production of pro-inflammatory cytokines. Furthermore, the compound effectively downregulated the phosphorylation of NF-κB and IκBα. These results indicate that chebulinic acid (**51**) may mitigate the inflammatory damage to primary osteoblast cells through the inhibition of the NF-κB signaling pathway. This study underscores the potential of chebulinic acid (**51**) as a therapeutic agent, warranting further investigation into its mechanisms and efficacy in the context of inflammatory bone diseases [62].

The investigation revealed that corilagin (**22**) significantly mitigates pyroptosis by targeting the TIR domain of MyD88 and interacting with the CARD domain of ASC in macrophages. This action is crucial for defending against LPS-induced sepsis. The study provides valuable insights into the mechanisms by which corilagin exerts its protective effects, potentially guiding therapeutic strategies for sepsis treatment [63]. Another study demonstrated that corilagin (**22**) effectively mitigates atherosclerosis by inhibiting M1 macrophage polarization and inflammation. This outcome is achieved through the suppression of the TLR4-NFκB/MAPK signaling pathway. This research provides valuable insights into the molecular mechanisms by which corilagin exerts its therapeutic effects, offering potential strategies for the treatment of atherosclerosis [64].

Gallic acid (**68**) has been demonstrated to have significant anti-inflammatory capabilities, primarily evidenced by its effectiveness in attenuating the activity of the NF-κB pathway within LPS-stimulated RAW264.7 macrophages [65]. This modulation results in a marked reduction in inflammatory mediators including NO, IL-6, and TNF-α. Furthermore, in vivo studies utilizing a mouse model of experimental dry eye have shown that gallic acid (**68**) not only prevents and inhibits the apoptosis of corneal epithelial cells but also diminishes the levels of inflammatory agents in both the cornea and conjunctiva, while concurrently preserving the integrity of goblet cells. These findings suggest that gallic acid (**68**) could be a potent agent in the management of inflammation and associated pathological conditions [65].

### 4.3. Antiviral Activity

Numerous studies have reported the inhibitory effects of CF on viral infections caused by severe acute respiratory syndrome coronavirus 2 (SARS-CoV-2), influenza A virus (IAV), herpes simplex virus-1 (HSV-1), and human immunodeficiency virus type 1 (HIV-1).

The emergence of the zoonotic SARS-CoV-2, coupled with the subsequent coronavirus disease 2019 (COVID-19) pandemic, has had a profound impact on society. The swift propagation and ongoing emergence of new variants of SARS-CoV-2 pose a persistent threat to public health worldwide [66]. Given the widespread use of traditional Chinese medicine in the battle against COVID-19, this approach has served as a formidable tool in safeguarding public health [67]. Several effective Chinese patent medicines featuring CF as the primary ingredient have been endorsed by the National Medical Products Administration (NMPA) and the State Council’s Joint Prevention and Control Mechanism. These include Tibetan medicine influenza pills, cuitang granules, and qingyan dropping pills, which are noted for their ability to inhibit SARS-CoV-2. In a recent study, the aqueous extracts of CF have demonstrated promising potential as an effective therapeutic agent against SARS-CoV-2 [68]. This efficacy can be primarily attributed to the inhibition of 3-chymotrypsin-like cysteine protease (3CLpro), a critical enzyme necessary for the replication of the virus. The findings suggest that these extracts interfere with protease activity, thereby impeding the viral replication cycle [68]. One study observed inhibitory effects of two broad-spectrum antiviral natural products, chebulagic acid (**50**) and punicalagin (**38**), on the replication of SARS-CoV-2. Studies have shown that both chebulagic acid (**50**) and punicalagin (**38**), at noncytotoxic concentrations, can reduce virus-induced plaque formation in Vero E6 monolayers. These compounds appear to function as allosteric regulators, targeting the enzymatic activity of the viral 3CLpro [69]. The inhibitory activities of 1,2,3,4,6-penta-*O*-galloyl-*β*-D-glucose (**14**) against SARS-CoV-2 3CLpro demonstrate its potential for impeding viral protease activity, thereby underscoring its therapeutic potential for the treatment of SARS-CoV-2 infections [70]. RNA-dependent RNA polymerase (RdRp) is a prime target for antiviral therapies, particularly due to its central role in the replication of RNA viruses. However, the efficacy of nucleoside analog inhibitors against RdRp is frequently undermined by the proofreading capabilities inherent to coronaviruses. Recent studies have identified corilagin, a non-nucleoside inhibitor, as an effective agent targeting the SARS-CoV-2 RdRp. Corilagin (**22**) binds directly to RdRp, robustly inhibiting its polymerase activity, as evidenced by both cell-free and cell-based assays [71]. The compounds containing CF exhibit potential as inhibitors of SARS-CoV-2 3CLpro and RdRp, thereby impeding viral replication. Nonetheless, additional in vivo studies are essential to ascertain their safety and efficacy.

Influenza A virus (IAV) is responsible for both regular seasonal epidemics and sporadic yet severe pandemics, posing significant public health challenges. The study demonstrated that extracts containing CF, punicalagin (**38**), and ellagic acid (**76**) effectively inhibited the release of the H1N1 virus and mitigated the cytopathic effects associated with it. Further investigations confirmed the mechanism of action, revealing that punicalagin and ellagic acid interact with the neuraminidase (NA) enzyme, leading to a reduction in NA activity. This interaction underscores the therapeutic potential of these compounds in managing H1N1 infections [72]. Recent research has highlighted chebulagic acid (**50**) and chebulinic acid (**51**) as novel inhibitors capable of thwarting IAV replication. These compounds emerge as promising NA inhibitors, with potential for development into effective antiviral agents targeting IAV [73].

Previous studies have reported that CF extract or its active compounds had a good inhibitory effect on viruses such as HSV-1, HSV-2, and HIV-1. Two hydrolyzable tannins, punicalagin (**38**) and chebulagic acid (**50**), were isolated from CF. These compounds effectively inhibit the entry of HSV-1 into A549 cells by targeting and neutralizing key viral components. Additionally, in BALB/c rats infected with respiratory syncytial virus, these tannins significantly mitigate lung lesions and reduce viral burdens and inflammatory markers, including inducible nitric oxide synthase (iNOS), cyclooxygenase-2 (COX-2), and prostaglandin E2 (PGE2) [74]. An investigation demonstrated that chebulinic acid (**51**) and chebulagic acid (**50**), extracted from CF, exhibit potent antiviral effects against HSV-2. These compounds effectively inhibit the attachment and penetration of the virus into host cells, surpassing the efficacy of acyclovir, the conventional drug used for managing HSV infections [75]. The study reported that CF contains four inhibitors of HIV-1 integrase, specifically gallic acid (**68**) and three derivatives of galloyl glucose. Notably, these galloyl derivatives are crucial in inhibiting the 3′-processing activity of HIV-1 integrase [76].

### 4.4. Anticancer Activity

Cancer, characterized by the unregulated proliferation of cells, is a critical health issue globally. This disease progresses as cells grow abnormally and uncontrollably, eventually leading to terminal stages. Annually, cancer is responsible for approximately 9.8 million deaths, positioning it as the world’s second most prevalent cause of death [77]. In recent advancements, numerous novel methodologies have been formulated to address human malignancies. However, an unequivocally effective cure remains elusive. The exploration of natural products as potential anticancer agents represents a promising avenue. The extract of CF showed significant anticancer effects on five cancer cell lines, including breast cancer, colon cancer, melanoma, prostate cancer, and lung cancer, both in vitro or in vivo. A study highlights the ethanolic extract’s potent anticancer effects, particularly against breast cancer cells, through mechanisms including HDAC inhibition and antioxidative stress reduction. The extract was effective at 300 μg/mL and showed no adverse effects on normal organ function, suggesting its potential as a safe therapeutic option in cancer treatment [78]. In another study presented, the methanolic extract from CF demonstrated potent in vitro anticancer activity, exhibiting cytotoxic effects ranging from 75% to 95% across four human cancer cell lines derived from colon, melanoma, prostate, and lung cancers. This significant inhibition suggests the extract’s potential as a source for novel anticancer compounds.

Several studies have indicated that corilagin (**22**), gallic acid (**68**), and ellagic acid (**76**) isolated from CF possess potential anti-breast cancer activity. Breast cancer is a major disease significantly impacting women’s health, with its prevalence and intensity increasing globally [79]. In a study, it has demonstrated that corilagin (**22**) enhances both autophagy and the production of ROS, triggering apoptosis in breast cancer cell lines, specifically MDA-MB-231 and MCF-7. Notably, corilagin (**22**) was found to escalate ROS levels significantly in MCF-7 cells. However, when used in conjunction with N-acetyl-L-cysteine, a contrasting effect was observed, with a substantial reduction in ROS levels, which consequently inhibited cell death. The findings suggest that corilagin has the potential to inhibit breast cancer progression through mechanisms involving ROS-mediated apoptosis and autophagy. Therefore, corilagin (**22**) emerges as a potential novel anticancer agent for the treatment of breast cancer, which contributes to the growing body of evidence supporting the therapeutic properties of phytochemicals in oncology [80]. Gallic acid (**68**) was found to significantly inhibit the viability of acidity-adapted MCF7 cells (MCF7-6.4/12w) at concentrations exceeding 30 µM, while at lower concentrations (5–20 µM), it diminished the metastatic features promoted by acidic environments. Furthermore, this compound effectively downregulated the PI3K/Akt signaling pathway and reduced the nuclear localization of *β*-catenin, both of which were upregulated in the MCF7-6.4/12w cells. These observations suggest that gallic acid (**68**) holds considerable promise as a therapeutic candidate for targeting metastatic characteristics in breast cancer cells exposed to acidic conditions [81]. Another study demonstrated that gallic acid (**68**) effectively diminished the activity of the exosomal secretory pathway in breast cancer cell lines, underscoring its potential as an anticancer agent. This finding contributes to the understanding of gallic acid’s (**68**) mechanism of action in inhibiting cancer progression [82]. In an analysis of ellagic acid’s (**76**) therapeutic potential, it has been observed that this compound effectively diminishes the colonization of cancer cells and promotes apoptosis. Additionally, the treatment with ellagic acid (**76**) results in a notable downregulation of CDK6 expression in human breast cancer cell lines. This investigation underscored ellagic acid’s capability as a significant inhibitor of CDK6, warranting further exploration within the realm of CDK6-targeted anticancer strategies.

Other studies have shown that 1,2,3,4,6-penta-*O*-galloyl-*β*-D-glucose (**14**) effectively induces apoptosis in HepG2 cells. This apoptotic effect is mediated through the activation of the p53 signaling pathway, contributing to its anti-hepatocellular carcinoma properties in vitro. This finding is significant as it underscores the potential therapeutic role of 1,2,3,4,6-penta-*O*-galloyl-*β*-D-glucose in treating liver cancer, warranting further investigation [83]. In another study, RT-PCR and western blot techniques were utilized to demonstrate that corilagin (**22**) possesses anti-cervical cancer properties by promoting apoptosis in tumor tissues via both the PI3K/AKT and MAPK signaling pathways. Consequently, this study offers a theoretical foundation for further research on corilagin as a potential bio-resource in the development of anti-cervical cancer drugs and functional foods [84]. Chebulagic acid (**50**) effectively inhibited the expression of AURKA as well as the AURKA/*β*-catenin/Wnt signaling pathway, both in vitro and in vivo. The findings collectively indicate that high levels of AURKA expression could serve as an independent predictor of poor prognosis in gastric cancer patients. Furthermore, chebulagic acid markedly suppressed the tumorigenic capabilities of gastric cancer cells and hindered the AURKA/*β*-catenin/Wnt pathway [85]. In summary, a comprehensive body of evidence suggests that CF, a renowned traditional herb, exhibits potential as a novel therapeutic option for multiple cancer types.

### 4.5. Antibacterial Activity

The reported antibacterial activity of CF spans a broad spectrum, effectively targeting various pathogenic Gram-positive and Gram-negative bacteria. Extracts from CF have been shown to have antibacterial effects on several bacterial strains. It effectively inhibits *Helicobacter pylori*, *Xanthomonas*, *Salmonella typhi*, *Staphylococcus epidermidis*, *Staphylococcus aureus*, *Bacillus subtilis*, *Pseudomonas aeruginosa*, and *Streptococcus mutans* in accordance with the extraction methods [86]. Moreover, in a study, ethanolic extract of CF demonstrated effective antibacterial activity against *Enterococcus faecalis*. Additionally, it was observed that the antibacterial efficacy of the extract increased proportionally with its concentration until reaching a saturation point [87]. The research findings indicate that ethyl acetate, methanol, and aqueous extracts from CF all possess inhibitory effects on *Porphyromonas gingivalis*. Additionally, ethyl acetate extract contained a higher diversity of phytochemicals and exhibited superior anti-*P. gingivalis* activity relative to the methanol and aqueous extracts [88]. A separate study demonstrated that ethanol extract of CF possesses potent anticaries properties by inhibiting glucan formation and the expression of associated genes, while also exhibiting anti-*Streptococcus mutans* effects at concentrations that are minimally cytotoxic. Consequently, this ethanol extract of CF holds potential for incorporation into oral hygiene products aimed at managing dental caries due to its antibacterial capabilities. Additional research is needed to identify the specific compounds responsible for its antibacterial activity [44]. The extract of CF and its constituent phenolic acid, and corilagin (**22**), both demonstrate antibacterial properties against *Staphylococcus aureus* by inhibiting biofilm formation. The half-maximal inhibitory concentrations for the extract and corilagin (**22**) were found to be 0.13 µg/mL and 3.18 µg/mL, respectively. The potential underlying mechanism of action could involve the downregulation of gene transcription associated with quorum sensing pathways, specifically targeting genes such as staphylococcal accessory regulator A, intercellular adhesion accessory gene regulator A, and RNAIII. These results suggest that CF extract, particularly due to the activity of corilagin (**22**), might serve as an effective antibacterial agent [89]. This research has demonstrated the effectiveness of ellagic acid (**76**) in combating significant and commonly resistant pathogenic bacteria, including *Methicillin-resistant Staphylococcus aureus*, *Pseudomonas aeruginosa*, and *Escherichia coli*. Additional investigations are warranted to explore the potential of ellagic acid as a therapeutic agent in pharmaceutical applications [90]. It is important to note that although studies on the antibacterial activity of CF are promising, the specific mechanism of its antibacterial activity and its efficacy in practical clinical applications need to be verified and confirmed by more in-depth scientific studies and clinical trials.

### 4.6. Hepatoprotective Activity

Liver injury, often referred to as hepatotoxicity, poses a significant health threat as it can cause irreversible damage to this vital organ, potentially leading to severe conditions such as liver cirrhosis or liver failure. The use of natural medicinal plants is pivotal in contemporary drug discovery, given their vast potential as treatment sources. In light of the severe circumstances faced by individuals with liver toxicity worldwide, numerous plants have been identified for their therapeutic properties. Among these, triphala, a renowned and potent traditional Ayurvedic medicine from India, stands out due to its unique hepatoprotective and antioxidant characteristics. Triphala is a critical herbal blend in Ayurveda, the traditional Indian medical system, consisting of three medicinal fruits: *Terminalia chebula* Retz., *Phyllanthus emblica* Linn., and *Terminalia belerica* Retz. This composition underscores its importance and effectiveness in managing liver health issues [91]. CF water extract effectively shields the liver from acute and severe damage. The potential mechanisms underlying this protective effect likely include the boosting of antioxidant capabilities and the regulation of inflammatory responses [92]. In a study, pretreatment with chebulinic acid (**51**) was found to mitigate t-BHP-induced damage in L-02 hepatocytes. This protective effect is primarily achieved by inhibiting the production of ROS, reducing lactate dehydrogenase (LDH) levels, and boosting the expression of HO-1 and NADPH quinone dehydrogenase 1 (NQO1) through the MAPK/Nrf2 signaling pathway. In related animal studies, chebulinic acid significantly shielded mice from liver damage induced by carbon tetrachloride (CCl_4_). This was evidenced by decreased levels of alanine aminotransferase (ALT), aspartate aminotransferase (AST), and malondialdehyde (MDA), along with increased superoxide dismutase (SOD) activity, improved liver histopathology, and activation of the Nrf2/HO-1 signaling pathway. Studies conducted on cells and in animal models consistently demonstrated and elaborated the hepatoprotective effects of chebulinic acid (**51**) [4]. 1,2,3,4,6-penta-*O*-galloyl-*β*-D-glucose (**14**) effectively mitigates hepatic steatosis induced by a high-fat diet and counteracts the diet-induced changes in gene expression related to lipid metabolism. Demonstrated to be well tolerated, 1,2,3,4,6-penta-*O*-galloyl-*β*-D-glucose (**14**) holds promise as a potential treatment for non-alcoholic fatty liver disease [93].

### 4.7. Nephroprotection Activity

CF is documented in the Four Medical Tantras for its efficacy in treating Mikpa, referred to as “HuangShui” in Tibetan medicine, a condition linked with renal dysfunction. Its therapeutic properties include alleviation of swelling and enhancement of kidney health. Moreover, the “Chinese Materia Medica-Tibetan Medicine Scroll” highlights its role in enhancing digestion and addressing renal deficiencies. Additionally, various formulations from the Four Medical Tantras and the Standard of Tibetan Medicine, like “ShiWeiHeZiWan” and “TongFengTangSan”, are extensively utilized in managing inflammatory renal and joint disorders including nephritis, renal failure, hematuria, gout, and arthritis [94,95]. Modern pharmacological research corroborates the effectiveness traditionally attributed to CF, demonstrating the potent antioxidant and anti-inflammatory properties that underlie its nephroprotective benefits [96]. Aqueous extract of CF demonstrates protective effects on hypertensive nephropathy by attenuating inflammation and enhancing fibrotic repair. This is achieved through the modulation of the TLR4/MyD88/NF-κB signaling pathway, reinforcing its traditional application in the treatment of hypertensive nephropathy and related chronic kidney conditions [97]. The primary constituents of CF, such as corilagin (**22**), chebulinic acid (**51**), chebulagic acid (**50**), chebulic acid (**81**), gallic acid (**68**), and ellagic acid (**76**), have been reported to ameliorate renal histological damage in rats. This improvement is associated with a reduction in pro-inflammatory cytokines including TNF-α, IL-1, and IL-6 [98].

### 4.8. Neuroprotective Activity

Extract from CF demonstrates promising neuroprotective properties, offering protection against neuronal damage in experimental ischemic stroke models and reducing toxicity induced by A*β* aggregation. Additionally, it has been shown to enhance memory functions in cases of impairment [99,100,101]. In research, it was observed that rats treated with haloperidol displayed behavioral disruptions, oxidative stress, and inflammation. However, administering aqueous extract of CF significantly ameliorated these issues. This extract demonstrated both antioxidant and anti-inflammatory effects and helped in preserving the integrity of neuronal structures. These findings point to CF as a potential therapeutic option for counteracting drug-induced neurotoxicity. The neuroprotective effects of CF appear to derive from several mechanisms. Primarily, it acts as an antioxidant, neutralizing free radicals and diminishing oxidative stress. Additionally, it inhibits the production of pro-inflammatory cytokines, showcasing anti-inflammatory effects. Lastly, it contributes to the maintenance and repair of neuronal pathways [102]. In a separate study, the ability of CF extract to protect against cerebral ischemia–reperfusion damage was evidenced through the augmentation of antioxidant enzyme activities and the increased nuclear translocation of nuclear factor erythroid 2-related factor 2 (Nrf2), along with the upregulation of antioxidant proteins. Concurrently, there was a noticeable decrease in both cell apoptosis and levels of reactive oxygen species. Furthermore, CF provides neuroprotection by activating the Nrf2 signaling pathway, which in turn suppresses apoptosis [103].

### 4.9. Anti-Diabetic Activity

Diabetes, an endocrine disorder characterized by insufficient insulin production, often leads to chronic hyperglycemia. Notably, obesity is a primary contributor to the development of type 2 diabetes [104]. Research demonstrates that extract derived from CF significantly contributes to managing type 2 diabetes. Aqueous extract from CF was found to reduce oxidative stress and enhance the expression of SIRT1 in diabetic rats, indicating a notable anti-diabetic effect [105]. Studies have demonstrated that higher doses of CF (600 mg/kg) exhibit significant therapeutic potential, particularly showing enhanced anti-diabetic and antilipidemic effects. Additionally, these doses offer pronounced hepatoprotective and renoprotective benefits compared to lower doses. Utilizing CF, either as a standalone treatment or adjunctive to traditional anti-diabetic medications, holds promise as an innovative approach in diabetes management [106]. Chebulinic acid (**51**) extracted from CF has been identified as a promising anti-diabetic agent that simultaneously targets PTPN9 and PTPN11. The results suggest that chebulinic acid acts as a dual allosteric inhibitor, exhibiting strong binding affinities with IC_50_ values of 34 nM for PTPN9 and 37 nM for PTPN11. The inhibitor showed a slow but synergistic interaction when binding to both enzymes. Furthermore, chebulinic acid enhanced glucose uptake in differentiated 3T3-L1 adipocytes and C2C12 muscle cells by activating the AMPK signaling pathway. These observations support the potential of chebulinic acid as a therapeutic candidate for the treatment of type 2 diabetes.

### 4.10. Other Activities

In addition to the above pharmacological effects, CF extract was an effective pharmaceutical agent for protecting the skin against photodamage [107]. CF showed remarkable anticonvulsant effects and enhanced the efficacy of subtherapeutic doses of phenytoin and valproate, suggesting its potential role as an adjunctive therapy in epilepsy. This could be particularly beneficial in mitigating the cognitive decline and oxidative stress typically associated with antiepileptic drugs [108]. Furthermore, CF has demonstrated potential effectiveness in managing IgE-dependent conditions like bronchial asthma and atopic dermatitis [109].

**Table 2:** Pharmacological activities of extracts and compounds from Chebulae Fructus.

Biological Activities	Extracts/Compounds Dose	Assays	Subjects	Mechanisms/Effects	Ref.
**Antioxidant activity**					
	70% methanol extract	reducing power assay, DPPH and nitric oxide radical scavenging assay	In vitro	reduce oxidative stress	[19]
	70% methanol extract (10, 50 and 100 mg/kg)	in vivo antioxidant assay, in vitro antioxidant assay	In vivo/In vitro	scavenge various free radical	[41]
	water, methanol, 95% ethanol extract	chemiluminescence assay	In vitro	scavenge ROS levels	[42]
	70% aqueous ethanol extract (10, 25, 50, 100 mg/mL)	liver lipid peroxidation assay, DPPH radical scavenging assay	In vivo/In vitro	inhibit lipid peroxidation	[43]
	70% ethanol extract	DPPH and ABTS radical scavenging activity, MTT assay	In vitro	scavenge various free radical	[44]
	70% methanol extract (50–500 μg/mL)	DPPH, nitric oxide and hydrogen peroxide radical scavenging assay	In vitro	scavenge ROS levels	[45]
	chebulagic acid (**50**), chebulinic acid (**51**)	FRAP determination, DPPH and ABTS^+^ inhibition determination, LDH and CCK-8 determinations, UHPLC–ESI-Q-TOF-MS analysis	In vitro	inhibit ROS scavenging and iron chelation	[48]
	gallic acid (**68**) (20 mg/kg)	metabolic syndrome in rats	In vivo	reduce oxidative stress	[50]
	ellagic acid (**76**)	ABTS and DPPH radical scavenging activity assay, hydroxyl radical activity assay	In vitro	scavenge various free radical	[51]
**Anti-inflammatory activity**					
	aqueous extract (30, 100, and 300 mg/kg)	Western blot, RT-PCR, ELISA, luciferase assay	In vivo/In vitro	regulate anti-inflammatory factors in vivo and suppress STAT1/3 and NF-ĸB signaling in vitro	[53]
	aqueous extract (5, 10, and 20 µg/mL	MTT, Western blot, histological examination of joints	In vivo/In vitro	inhibit NF-κB/MAPK signaling pathway	[54]
	ethanol extract (150 and 300 mg/kg)	carrageenan-induced paw edema in rat	In vivo	inhibit carrageenan-induced rat paw edema	[43]
	ethyl acetate extract	Western blot, RT-PCR	In vivo	inhibit TLR4, MyD88, NF-κB signaling pathways	[55]
	chebulagic acid (**50**), chebulanin (**44**), corilagin (**22**)	CCK-8, ELISA	In vitro	reduce levels of IL-6 and IL-8	[56]
	chebulanin (**44**) (25, 50, and 100 μM)	ELISA, Western blot, immunofluorescence staining	In vivo/In vitro	reduce levels of IL-6 and TNF-*α*, inhibit NF-κB/MAPK signaling pathways	[58]
	corilagin (**22**) (6.25 and 12.5 μM)	CCK-8, ELISA, Western blot, immunofluorescence staining, flow cytometry, RT-PCR	In vivo/In vitro	inhibit NF-κB/MAPK signaling pathways	[59]
	1,2,3,4,6-penta-*O*-galloyl-*β*-D-glucose (**14**) (20, 40, and 80 μM)	CCK-8, Western blot, hematoxylin and eosin, ELISA	In vivo/In vitro	inhibit MAPK/NF-κB and ERK/Nrf2/HO-1 signaling pathways	[61]
	chebulagic acid (**50**) (5 and 10 mg/kg)	MTT, RT-PCR, Western blot, micro-CT scanning,	In vivo /In vitro	inhibit NF-κB signaling pathway	[62]
	corilagin (**22**) (12.5, 25, and 50 mg/kg)	Western blot, ELISA, cell death assay, SPR analysis, transcriptome analysis, molecular docking	In vivo	inhibit of NF-κB activation and NLRP3 production	[63]
	corilagin (**22**) (60, 120, and 240 μM)	MTT, RT-PCR, Western blot, molecular docking Immunofluorescence staining	In vivo/In vitro	inhibit TLR4-NFκB/MAPK signaling pathway	[64]
	gallic acid (**68**) (100 μM)	Western blot, ELISA, fluorescence assay	In vivo/In vitro	reduce levels of NO, IL-6 and TNF-*α*, inhibit NF-κB signaling pathway	[65]
**Antiviral activity**					
	aqueous extract	biomolecular interaction assay, enzyme kinetic study, molecular docking	In vitro	inhibit SARS-CoV-2 3CLpro activity	[68]
	chebulagic acid (**50**), punicalagin (**38**) (1.56–100 μM)	antiviral assay, cytotoxicity assay, enzymatic inhibition assay, molecular docking	In vitro	inhibit SARS-CoV-2 3CLpro activity	[69]
	1,2,3,4,6-penta-*O*-galloyl-*β*-D-glucose (**14**) (0–100 mM)	protease activity assay, dose–response curve analysis, molecular modeling	In vitro	inhibit SARS-CoV-2 3CLpro activity	[70]
	corilagin (**22**) (2.5, 10, and 40 μM)	molecular dynamic simulation, BLI binding assay, in vitro polymerase activity assay, CCK-8 assay, CPE assay	In vitro	inhibit SARS-CoV-2 RdRp activity	[71]
	50% ethanol aqueous extract (25, 50, 100 μg/mL), punicalagin (**38**) (12.5, 25, 50 μM), ellagic acid (**76**) (6.25, 12.5, 25 μM)	competitive inhibition assays, MTT assay, CPE assay, virus yield reduction assay	In vitro	inhibit IAV activity	[72]
	chebulinic acid (**51**), chebulagic acid (**50**)	one-cycle infection, inhibition assay virus, release inhibition assay, NA inhibition assay, viral yield reduction assay	In vitro	inhibit IAV replication	[73]
**Antibacterial activity**					
	ethanol extract (0, 5, 10, 15 and 20 μg/mL)	RT-PCR, colony forming unit, glucan formation, drug susceptibility test	In vitro	inhibit glucan formation and related gene expression	[44]
	75% ethanol extract and corilagin	hemolysis assay, Western blot, RT-PCR	In vitro	inhibit biofilm formation, quorum sensing, and toxin secretion	[89]
**Anticancer activity**					
	ethanol extract (250 and 500 mg/kg)	MTT, tumor parameters assay, histopathology	In vivo	inhibit breast cancer cell line (MCF-7)	[78]
	corilagin (**22**) (5, 15, 25 mg/kg)	immunofluorescence, flow cytometry, Western blot, xenograft assay	In vivo/In vitro	induce apoptosis and autophagy	[80]
	gallic acid (**68**) (25, 50, 75 and 100 μM)	RT-PCR, Western blot, cytotoxicity assay, apoptosis analysis	In vitro	inhibit PI3K/Akt signaling pathway	[81]
**Hepatoprotective activity**					
	aqueous extract (0, 50, 100 and 200 mg/kg)	serum biochemical analysis, histopathological, immunohistochemical	In vivo	reduce levels of TNF-*α*, IL-1*α* and IL-6	[92]
	chebulinic acid (**51**) (37.5, 75 and 150 mg/kg)	RT-PCR, Western blot, histopathology, immunohistochemical	In vivo/In vitro	inhibit MAPK/Nrf2 and activate of Nrf2/HO-1 signaling pathway	[4]
	1,2,3,4,6-penta-*O*-galloyl-*β*-D-glucose (**14**) (25 and 300 mg/kg)	serum biochemical assay, PT-PCR, Western blot	In vivo	reduce protein expression of CD36	[93]
**Nephroprotective activity**					
	aqueous extract (30, 60 and 90 mg/kg)	RT-PCR, Western blot, Network pharmacology, ELISA, histological staining	In vivo/In vitro	regulate TLR4/MyD88/NF-κB axis	[97]
**Neuroprotective activity**					
	50% methanol extract (200 mg/kg)	CCK-8, flow cytometer, immunofluorescence staining, Western blot	In vivo/In vitro	stimulate Nrf2 signaling pathway	[103]
**Anti-diabetic activity**					
	aqueous extract (500 and 1000 mg/kg)	immunohistochemistry, ELISA, histopathology	In vivo	decrease oxidative stress and increase the expression of SIRT1	[105]

## 5. Toxicity

Although CF has been widely utilized in clinical practice for many years, comprehensive evaluations of its safety and toxicity are still lacking. One significant advantage of CF in its potential development as an herbal functional food or nutraceutical product is the apparent absence of debilitating or toxic side effects. A study demonstrated that acute oral administration of CF (ranging from 250 to 2000 μg/mL) is relatively non-toxic in vitro. This finding provides practical guidance for selecting safe dosages for subsequent clinical trials [110]. In an investigation of oral toxicity, administration of a single 2000 mg/kg dose of the extract to rats resulted in neither fatalities nor any noticeable pathological changes in their internal organs. Furthermore, a subsequent 14-day study involving repeated oral doses revealed that the ethyl acetate-soluble fraction of CF ethanol extracts was well tolerated, with no adverse effects observed at dosages up to 2000 mg/kg [111]. In addition, the water extract of CF demonstrated no acute (LD_50_ > 5000 mg/kg) or chronic oral toxicity, indicating a high level of safety in rats. When these findings are projected onto human applications, it appears that CF water extract is safe for consumption at daily doses of 300, 600, and 1200 mg/kg [112].

The toxicity profile of CF was evaluated in a controlled study on rats. The study observed no significant toxic effects on the brain and stomach at any dose (200, 400, and 800 mg/kg), but slight lymphocyte hyperplasia in the spleen across all doses tested, suggesting some potential impact on the immune system. Overall, CF did not show major toxic effects, but caution is advised, especially regarding its effects on the spleen and possible implications for blood parameters at certain doses [113]. In another study, a tannin-rich fraction from CF did not exhibit any toxic symptoms or cause mortality in Wistar albino rats at a single dose of 5000 mg/kg, observed over 14 days. However, prolonged exposure at a lower dose (1000 mg/kg for 28 days) caused a marked reduction in body weight, food and water intake, and mild disturbances in liver and kidney functions, as indicated by increased levels of serum urea, glucose, and AST, and histopathological findings revealed granulomatous inflammation in the liver [114]. While the safety of CF is established, this herbal medicine is extensively utilized as both food and medicine across China and globally. However, the toxicological examinations of this herbal medicine remain insufficient. Consequently, numerous unresolved issues necessitate further investigation through additional in vivo studies and clinical trials to provide clearer insights.

## 6. Pharmacokinetics

Investigating the pharmacokinetic properties of chemical constituents in CF extract is crucial for elucidating the metabolic pathways of drugs once they are introduced into biological systems, thereby underpinning drug research and development. Given the intricate nature of the chemical compounds in traditional Chinese medicine extracts, it is essential to delineate how these substances integrate into the bloodstream to enhance our understanding of their pharmacokinetic characteristics. At present, a sophisticated ultraperformance liquid chromatography–tandem mass spectrometry (UPLC-MS/MS) technique has been developed to measure the plasma levels of nine active compounds (chebulic acid (**81**), gallic acid (**68**), protocatechuic acid (**63**), corilagin (**22**), chebulagic acid (**50**), chebulinic acid (**51**), 1,2,3,4,6-penta-*O*-galloyl-*β*-D-glucose (**14**), ellagic acid (**76**), and ethyl gallate (**73**)) following the oral administration of CF extracts in rats. This validated UPLC-MS/MS approach was successfully applied to the plasma pharmacokinetic analysis of the CF extracts. The pharmacokinetic data showed that chebulagic acid, in particular, exhibited a significant plasma exposure, as evidenced by its high area under the concentration-time curve (AUC_(0-tn)_, 231,112.38 ± 64,555.20 h ng/mL) and peak concentration (Cmax, 4983.57 ± 1721.53 ng/mL). In addition, the elimination half-lives (T_1/2_) for chebulinic acid (**51**), corilagin (**22**), and chebulagic acid (**50**) were found to be 43.30, 26.39, and 19.98 h, respectively, indicating that these compounds are retained and slowly metabolized in the body for extended periods of time. The results of this study provide a theoretical basis for the future clinical use of CF extracts [115]. In a recent study examining the pharmacokinetics of chebulinic acid (**51**) in male SD rats, it was found that chebulinic acid (**51**) demonstrated relatively moderate oral bioavailability at 37.56 ± 7.3% when administered at a dosage of 100 mg/kg. The study also highlighted chebulinic acid’s (**51**) substantial plasma protein binding capacity and its varying distribution levels in blood plasma, depending on concentration. More importantly, the research identified that chebulinic acid significantly induces the activity of several cytochrome P450 enzymes in the liver, specifically CYP1A2, 2C11, 2D2, and 2E1. This induction was consistent with both in vitro and in vivo assessments, indicating reliable and significant effects on these liver enzymes after 14 days of chebulinic acid exposure. Such induction of cytochrome P450 enzymes could influence the metabolism of other drugs and potentially modify therapeutic outcomes. Overall, these findings suggest that chebulinic acid (**51**) not only possesses beneficial pharmacological properties but also exhibits a complex interaction with liver enzymes that could impact its therapeutic efficacy and drug interactions [116]. This collection of research illustrates how pharmacokinetic investigations aid in revealing the underlying pharmacodynamic principles of traditional Chinese medicine, while also offering directions for their clinical application. It is important to note that natural products often exhibit low water solubility and bioavailability. Future enhancements in drug bioavailability may be achieved by employing sophisticated drug delivery systems.

## 7. Conclusions and Future Perspectives

CF has been extensively utilized across various traditional medicine systems, including in Ayurvedic, Unani, and Tibetan traditional medicine, among others. In addition to their medicinal applications, CF is also valued for its nutritional benefits. To date, researchers have identified over 149 compounds within CF, predominantly consisting of tannins and phenolic acids. A vast array of studies have demonstrated that both the extract of CF and its individual compounds possess a range of biological properties, such as antioxidant, anti-inflammatory, antiviral, anticancer, antibacterial, hepatoprotective, nephroprotective, neuroprotective, and anti-diabetic effects, among others. It is believed that tannins and phenolic acids are the principal bioactive constituents responsible for the majority of these pharmacological activities.

Despite the extensive documentation in the literature confirming these effects, there remains a need for more detailed research to address specific gaps in our understanding of CF’s phytochemistry and pharmacology. Notably, CF is considered to be largely nontoxic and exhibits far fewer side effects than many conventional chemical drugs offering similar benefits. However, the research to date has primarily focused on the aqueous and alcoholic extracts of CF, with less attention given to its individual components. This has led to a lack of clarity regarding the material basis of CF’s pharmacological actions.

Historically, CF has been a component of numerous highly effective medicinal prescriptions [2,91]. Nonetheless, these formulations have not been rigorously studied with regard to their mechanisms of action. To better support clinical practitioners and enhance the therapeutic efficacy of formulations containing CF, it is crucial to deepen our understanding of the mechanisms of action, the material basis, and their interrelationships. Furthermore, the molecular mechanisms underlying the pharmacokinetics of CF have not been sufficiently explored, complicating the analysis of its pharmacological mechanisms. Urgent and focused research in this area is essential for advancing the application and our understanding of CF.

In conclusion, this detailed review aims to provide a comprehensive discussion on the traditional uses, phytochemistry, pharmacological properties, toxicity, and pharmacokinetics of CF. It is anticipated that this review will furnish valuable insights to researchers, facilitating further development and utilization of CF in various therapeutic contexts.

## Data Availability

Data are contained within the article.

## Abbreviations

CF	Chebulae Fructus
DPPH	2,2-diphenyl-1picryl-hydrazyl-hydrate
ABTS	2,2′-azino-bis (3-ethylbenzothiazoline-6-sulfonic acid)
FRAP	Fe^3+^-reducing antioxidant power
LDH	lactate dehydrogenase
NA	neuraminidase
LPS	lipopolysaccharide
NF-κB	Nuclear factor-κB
TNF-α	tumor necrosis factor-*α*
IL	interleukin
NLRP3	NOD-like receptor protein 3
iNOS	inducible nitric oxide synthase
COX-2	cyclooxygenase-2
PGE2	prostaglandin E2
IC_50_	half maximal inhibitory concentration
MAPK	mitogen activated protein kinase
NO	nitric oxide
ELISA	enzyme-linked immunosorbent assay
ROS	reactive oxygen species
TLR4	toll-like receptor 4
JNK	c-Jun N-terminal protein kinase
CCK-8	cell counting kit-8
RT-PCR	real-time polymerase chain reaction
A*β*	amyloid-*β*
ERK	extracellular regulated protein kinase
HO-1	hemeoxygenase-1
MMPs	matrix metalloproteinases
MIA	monosodium iodoacetate
SARS-CoV-2	severe acute respiratory syndrome coronavirus 2
IAV	influenza A virus
HSV-1	herpes simplex virus-1
NMPA	national medical products administration
RdRp	RNA-dependent RNA polymerase
3CLpro	3-chymotrypsin-like cysteine protease
PGE2	prostaglandin E2
CCl4	carbon tetrachloride
ALT	alanine aminotransferase
AST	aspartate aminotransferase
Nrf2	nuclear factor erythroid 2-related factor 2
AMPK	AMP-activated protein kinase
LD_50_	median lethal dose
UPLC-MS/MS	ultraperformance liquid chromatography-tandem mass spectrometry
